# Good death for people living with dementia: a qualitative study

**DOI:** 10.1186/s12877-023-04395-y

**Published:** 2023-10-16

**Authors:** Md Razib Mamun, Yoshihisa Hirakawa, KM Saif-Ur-Rahman, Young Jae Hong, Zean Song, Yuko Yoshida, Hiroshi Yatsuya

**Affiliations:** 1 Department of Public Health and Health Systems, Graduate School of Medicine, Nagoya University, Nagoya, Japan; 2 Health Systems and Population Studies Division, icddrb, Dhaka, Bangladesh; 3 College of Medicine, Nursing and Health Sciences, University of Galway, Galway, Ireland; 4 Evidence Synthesis Ireland and Cochrane Ireland, University of Galway, Galway, Ireland

**Keywords:** Dementia, Good death, Older people, End-of-life, Qualitative research

## Abstract

**Background:**

Ensuring a good death is one of the primary objectives of palliative care and end-of-life care. There is insufficient evidence regarding what defines a good death for people living with dementia. Obtaining an understanding of what constitutes a good death could help improve dementia care. This study aimed to explore how multiple stakeholders perceive a good death for people living with dementia.

**Methods:**

This qualitative study was carried out across six prefectures in Japan. Enrollment of participants took place within dementia outpatient clinics, hospitals, daycare centers, and community centers. A total of thirty-three in-depth interviews with people living with dementia, physicians, and nurses were conducted. Six focus group discussions were performed with family caregivers and care workers. Verbatim transcripts of the interviews were prepared, and inductive content analysis was used to examine the data.

**Findings:**

Regarding the perception of a good death, the following themes were derived: (1) painless death; (2) dying in a preferred environment; (3) family’s coping with loss; (4) maintaining regular life; (5) living with respect; and (6) preparation for death. All these themes are interrelated. Participants viewed a good death as a process rather than a single event.

**Conclusion:**

This study identifies crucial components of a good death for people living with dementia. The findings could be used to improve dementia care.

**Supplementary Information:**

The online version contains supplementary material available at 10.1186/s12877-023-04395-y.

## Background

It is estimated that by 2050, approximately 152 million individuals worldwide will be affected by dementia [1]. In Japan, estimates suggest that approximately 7 million people aged ≥ 65 years will have dementia by 2025 [2]. People living with dementia (PLWD) experience deterioration of their memory, judgment, reasoning ability, and capability to care for themselves over time. It is only in the final stage that PLWD lose their ability to respond, converse, and control their movement. With the rise in the number of PLWD globally, palliative care and end-of-life (EoL) care have become increasingly vital in dementia care. Although palliative care also involves end-of-life care, the main difference is that palliative care can be applied at any stage of the disease. Attaining a good death is a core objective of both palliative care and EoL care.

The importance of palliative care to enhance the quality of life for those facing life-threatening illnesses and their families has been widely emphasized. The World Health Organization (WHO) defines palliative care as “an approach to improve the quality of life for patients and their families dealing with the challenges of life-threatening illnesses” [3]. While traditionally focused on cancer patients, palliative care has also been applied to dementia care [4–6]. It is relevant at any stage after a dementia diagnosis and can be provided for any duration. The European Association of Palliative Care (EAPC) outlined the key principles of palliative care in dementia in a white paper. These principles include consistent, person-centered care, collaborative decision-making, establishing care preferences and advance care planning, ensuring essential comfort and symptom control, offering psychosocial and spiritual assistance, and early identification of the dying phase [4]. EoL care aims to support individuals in the advanced stages of life-restricting conditions, ensuring their comfort until death while respecting their preferences. A recent systematic review explored how EoL in PLWD was measured and conceptualized. The review highlighted mixed reports on how EoL was defined, focusing on the needs of PLWD rather than on defining EoL due to the complexity of such a definition [7].

An essential goal of palliative care and end-of-life care would be a good death, which has been regarded as a series of events rather than a single event [8–10]. According to the Institute of Medicine, USA, a good death is “free from avoidable distress and suffering for the patient, family, and caregivers, in general accord with the patient’s and family’s wishes, and reasonably consistent with clinical, cultural, and ethical standards” [11]. Although death is mainly a biological event, both death and the perception of a good death are imbued with social and cultural aspects as well as individual beliefs [9, 12]. However, there is a considerable lack of evidence on the perception of a good death for PLWD. Several systematic reviews have been conducted to summarize the concepts of a good death from the perspective of patients, family members, physicians, and nurses, but the majority of the studies have been limited to the context of cancer and cardiovascular disease [9, 13, 14]. Nevertheless, those systematic reviews highlighted not only the clinical but also the psychological and social components of a good death. Complexity regarding the perceptions concerning good death inherently exists due to the differences in personal perspective, sociocultural context, and the types and patterns of disease [9, 10, 15].

A good death in PLWD was previously reported mostly from the viewpoints of family members and health service providers, and physical comfort, spirituality, person-centered communication, being accompanied by familiar objects and individuals, and treatment preferences have been reported as components of a good death [16–18]. A scoping review discovered that the voice of PLWD was not considered when exploring the challenges of implementing palliative care in dementia [19]. There might be differences in their views from those of different stakeholders, including family caregivers and health service providers.

To enhance dementia care, understanding what constitutes a good death for PLWD is crucial, as it informs improved palliative and end-of-life care. We intended to explore the perception of a good death for PLWD among PLWD as well as among family caregivers, physicians, nurses, and care workers. The findings from this study would be useful for family caregivers and health service providers in planning palliative care and EoL care for PLWD in alignment with the viewpoints of a good death.

## Methods

### Design and setting

This study employed a qualitative methodological approach. It was conducted across six prefectures (Hokkaido, Akita, Aichi, Gifu, Hyogo, and Fukuoka) in Japan and involved in-depth interviews (IDIs) and focus group discussions (FGDs) with PLWD, family caregivers, physicians, nurses, and care workers.

### Sampling strategy and data collection

In each prefecture, the lead researcher (Y. H.), a geriatrician, used his networks in Hokkaido, Akita, Aichi, Gifu, Hyogo, and Fukuoka prefectures to identify dementia outpatient clinics and daycare centers and then contacted the respective physicians and center managers. Those physicians and center managers assisted in identifying potential participants. We used convenience sampling to recruit participants. Interviews with participants were conducted in multiple dementia outpatient clinics, hospitals, daycare centers, and community centers from October 2019 to March 2020.

The sample size was determined based on the data saturation model, which was defined by agreement within the study team as the point at which new interviews offered no new information regarding the research objective compared to data obtained from prior interviews. We conducted in-depth interviews (IDIs) with PLWD, physicians, and nurses. These healthcare provider participants were assumed to have experiences in the care of PLWD until their death, namely end-of-life (EoL), although we did not collect detailed information regarding the experience. Noninstitutionalized (who were not hospitalized/in a long-term care facility) PLWD who visited dementia outpatient clinics were considered. PLWD who were deemed capable of giving consent by their family member were considered to participate in the study. None of the PLWD participants were living alone, and all lived with their families. Information regarding the stage of dementia was not recorded. However, their noninstitutionalized status and ability to provide consent, to a certain degree, suggest that none of them were likely to have a severe/advanced stage of dementia. Indeed, none had advanced-level dementia in the present study. Family caregivers were also recruited from the outpatient clinics but were not limited to those caring for the PLWD included in the present study. None of the family caregiver participants had a family member with advanced-level dementia. A total of thirty-three IDIs were conducted. IDIs were 30–40 min long. Focus group discussions (FGDs) were separated by participant type: family caregiver and care worker. The number of participants in each FGD ranged from 6 to 8. Six FGDs were conducted, and each FGD lasted approximately 60–70 min. For both IDIs and FGDs, data were collected using a topic guide (supplementary file 1) and performed by the lead researcher. The interviewer has expertise in qualitative research and dementia care. The interviewer strictly ensured that the focus remained on the participants’ experiences and viewpoints. The interviewer had no physician-patient relationship with the PLWD participants and had no prior relationship with any participant. While talking about the time of approaching death, the interviewer described it as the period when individuals are nearing the end of their lives or when they are facing the prospect of dying. For both IDIs and FGDs, the participants were asked about their views on a good death. Specifically, participants were asked about their opinion on what constitutes a “good death”, how a good death can be achieved, and which components are important to achieve a good death. Several probing questions were asked to obtain more information and clarification based on the initial response. Interviews and discussions were audio-recorded. Table 1 displays the characteristics of the participants.

**Table 1:** Characteristics of the participants

Variables	In-depth interview (IDI)			Focus group discussion (FGD)	
	PLWD	Physician	Nurse	Family caregiver	Care worker
No. of Participants	12	11	10	22	19
Range of Participants per Focus Group				6–8	6–7
No. of Focus Group Discussions				3	3
Gender					
Female	8	2	10	19	17
Male	4	9		3	2
Age					
**<** 50		2	10	12	13
50–59		8		3	6
60–69		1		5	
70–79	2			2	
80–89	7				
≥ 90	3				

### Data analysis

Verbatim transcription of recordings was initially performed in Japanese, and then two bilingual research assistants (undergraduate students of medicine) translated it into English. To ensure the accuracy of the translation, we implemented a rigorous translation process. Both bilingual research assistants were native Japanese speakers and fluent in English. They were selected based on their excellent English proficiency, which was evaluated through their academic performance in English courses. Additionally, the lead researcher, with expertise in both languages, supervised the translation process, reviewing and refining the translated text to ensure fidelity to the original meaning. During the analysis phase, the translated versions of the transcripts were used. In this study, we employed content analysis [20]. The analysis in our study was conducted inductively. During the analysis phase, each transcript was extensively read by two researchers involved in the analysis. This helped researchers become familiar with the participants’ narratives. The analysis commenced with open coding, adopting a bottom-up approach devoid of preconceived frameworks. This method allowed themes to emerge directly from the data itself. Based on their commonalities, codes were divided into subcategories. Similar subcategories were grouped and identified as categories. Finally, themes were developed by summarizing categories. In the analysis, we followed the iterative process, and it was carried out until the major themes emerged.

### Trustworthiness

Triangulation, peer debriefing, and member checking were employed to achieve trustworthiness. Multiple data sources, analysts, and data collection methods were used to accomplish triangulation. The study team held debriefing meetings as needed where the interpretations of the codes, subcategories, categories, and themes were discussed and finalized upon consensus. Member-checking approach was applied, where interpretations of the findings were discussed with several participants to ensure that the researchers had accurately interpreted the participants’ perspectives.

### Ethical considerations

The Ethics Review Committee of Nagoya University Graduate School of Medicine (approval number 2015-04446984) approved this study. Before each interview, written informed consent was obtained from all participants. In addition to the consent of PLWD participants, proxy consent was obtained from respective family members. Interview transcripts were anonymized.

## Findings

This study revealed multiple aspects of a “good death” for PLWD. The qualitative content analysis resulted in six themes: painless death, dying in a preferred environment, family’s coping with loss, maintaining regular life, living with respect, and preparation for death. Our analysis revealed that all participant groups considered a “good death” to be not confined to a single event; it is a journey that unfolds over time, incorporating various elements and experiences.

Table 2 provides an overview of the themes and categories recognized by different participant groups involved in our study.

**Table 2:** Number of primary codes according to categories/subcategories recognized by different participant groups

Theme	Categories/sub-categories	Participant group				
		PLWD	Physician	Nurse	Family caregiver	Care worker
Painless death	Minimal suffering	14	14	12	24	18
	Avoidance of life-sustaining treatment	10			4	
	Inclination to rely on physicians’ opinions for the decision/ Shared decision-making regarding treatment preference		6	4	16	11
	Free from sadness	8	6	5	12	8
Dying in a preferred environment	Home as the preferred place	13				
	Health care facility as the preferred place				20	
	The preferred place may change			11		15
	Shared decision about the preferred place		4			4
	Presence of family member	16	11	13	15	14
	Health care facility as a home-like familiar environment				11	13
Family’s coping with loss	Family needs to accept the consequence		10	9	20	11
	Support of family member		12	13		12
Maintaining regular life	Engage in preferred activities	19	8	9	16	14
	Role of family in facilitating preferred activities		9	11	16	13
	Collaborative engagement in facilitating preferred activities		6	6	12	13
Living with respect	The desire of PLWD: not being a burden	13	4	5	17	10
	Treat PLWD the same as before		5	6	20	17
Preparation for death	Advance Care Planning (ACP)		18	15	19	15
	Complete unfinished task				8	

### Painless death

The first theme, ‘painless death’, refers to participants’ views on how their death could be a good one without experiencing physical and emotional pain. All participants expressed ‘painless death’ as a component of a good death. This theme includes minimal suffering, avoidance of life-sustaining treatment, and free from sadness. When discussing pain, participants used the terms ‘kutsuu’ and ‘itami’ in Japanese (In general, people may use both terms to express pain. ‘kutsuu’ may include aspects of total pain, such as physical, mental, social, and spiritual pain, and ‘itami’ may focus on the feeling of pain/discomfort due to having been hurt or illness). The elements within the theme of “painless death” varied among participant groups depending on their relationship with PLWD, particularly their opinions about the use of life-sustaining treatment.

#### Minimal suffering

Ensuring physical comfort was most frequently stated by the participants from all groups: A good death requires minimal suffering. This meant dying free of pain or other unpleasant symptoms.

*“I want to die as* Pinpinkorori*”.* (Pinpinkorori is a combination of Pinpin and Korori. Pinpin means staying healthy and independent in activities of daily living. Korori means instant death.) [PLWD, Female, ≥ 90 years].

The family caregivers reported that their loved ones have been experiencing various types of physical suffering due to dementia and other health complications. Previous knowledge of the death of a loved one with pain due to dementia and other health complications influenced the family caregiver’s views of a good death.

*“One of my neighbors died a few years back, he had dementia and couldn’t explain his pain (kutsuu), I am afraid that my husband may not be able to explain his pain (kutsuu) properly. However, I hope the physicians will take all measures to ease his pain (kutsuu).”* [Family caregiver, female, 60–69 years].

All physicians and nurses who participated in this study expressed that a painful death can never be a good death. Notably, their opinion regarding minimal suffering applied not only to a good death for PLWD but also to a good death in general.

*“In general, if anyone dies with enormous physical pain, do you think this is good? It is neither good for the patient nor for others. My job is to reduce the pain of dying patients.”* [Physician, Male, 50–59 years].

#### Life-sustaining treatment

While discussing more about minimal suffering, participants talked about life-sustaining treatment. Several PLWD participants mentioned their fear regarding life-sustaining treatment, especially mechanical ventilation. They believed that mechanical ventilation was painful and that they would not want it to be used for themselves. However, this did not mean indifference to receiving other medical treatment to avoid physical suffering, if necessary.

*“I told my daughter I would prefer not to use mechanical ventilation. However, I would like to take treatment to stop the pain (itami), if I have any because I don’t want to have pain (itami).”* [PLWD, Female, 80–89 years].

Family caregivers held divergent views. Some indicated their preference against life-sustaining treatment for their family members with dementia, which is consistent with the opinion of PLWD. However, the majority of family caregivers expressed a reliance on physicians to make decisions regarding the use of life-sustaining treatment, reflecting the concept of ‘inclination to rely on physicians’ opinions for the decision’.

Physicians and nurses expressed their opinions on life-sustaining treatment. However, the viewpoints of both groups were similar. Shared decision-making on current treatment options was emphasized by physician participants as important for balancing the benefits and burdens of any treatment under the overall condition of PLWD and aligning the treatment choice with their goals and preferences, which requires open discussions with PLWD and their families. In contrast, ‘advance care planning’ involves discussing future treatment preferences including PLWD and their family members. Nurses expressed similar opinions on the importance of shared decision-making, and both believed that shared decision-making in relation to treatment preference is necessary to ensure a good death. Care worker participants also emphasized shared decision-making regarding treatment preference.

#### Free from sadness

All participant groups recognized that the emotional state of the dying person greatly impacts their overall experience. They expressed the importance of emotional well-being and minimizing sadness, although they expressed their opinion in slightly different ways.

PLWD participants emphasized the importance of emotional comfort and reducing distress during their final stages, aiming for emotional happiness. Family caregivers also recognized the impact of sadness and aimed to create a supportive and loving environment for their loved ones, ensuring emotional comfort.

Physicians, nurses, and care workers echoed similar sentiments and emphasized the importance of addressing the emotional needs of PLWD rather than focusing only on physical health. They recognized the impact of emotional well-being on the overall quality of life.

*“Everyone needs to focus on reducing the physical and emotional pain* (Karada To Kokoro no Itami) *of people with dementia.”* [Care worker, Male, **<** 50 years].

Some care workers and physicians noted that emotional happiness is subjective and can be influenced by various factors, which may change over time. They stated the importance of understanding the unique preferences and needs of PLWD to promote emotional well-being. Family members, being closest, are seen as having insight into what brings emotional happiness or sadness to PLWD. By recognizing and addressing these individualized needs, they believed it was possible to ensure a death free from sadness. PLWD participants said that having family around and doing things they like brings them emotional happiness.

### Dying in a preferred environment

The theme “Dying in a preferred environment” encompasses various perspectives, as highlighted by the participants in our study. While the choice of preferred place and the presence of family members played a crucial role in the perception of the preferred environment, there were notable differences among PLWD and family caregivers, especially opinions regarding the preferred place.

The preferred place was explained from two different perspectives by PLWD and family caregiver participants. PLWD expressed a strong desire to face death in their familiar places, surrounded by their loved ones, as it was perceived by them as a good death.

*“Even if my condition changes suddenly and I die at home, I think it will be great because I want to live in my home until death.”* [PLWD, Male, 80–89 years].

In contrast, family caregivers often tended to favor healthcare facilities where their loved ones could receive continuous medical treatment until the end. The expected need for round-the-clock care and concerns about the deteriorating physical condition of PLWD influenced this preference.

*“I don’t want to see my father dying at home without any treatment. It would be good to die at a hospital, in a clean environment with the presence of a physician and family members.”* [Family caregiver, Female, **<** 50 years].

This discrepancy highlights a potential conflict between the preferences of PLWD and the viewpoints of family caregivers regarding the preferred place. While family caregivers expressed a preference for a place where PLWD can receive necessary medical treatment, the data from PLWD indicated home as a preferred place, even if their health condition deteriorates and they pass away there.

Nurses and care workers noticed a shift in family members’ preferences. Initially, they respected the wish of the dying PLWD to stay at home, but as the physical condition worsened, institutionalization was more likely to be considered. Perspectives on preferred places can change based on the physical conditions and availability of family caregivers. Few physicians mentioned shared decision-making with PLWD and family caregivers in determining the preferred place.

The presence of family members was universally acknowledged by all participant groups as a crucial component of the preferred environment. PLWD expressed a deep desire to have their loved ones, especially family members nearby, as death approached.

“*I think it is about seeing loved ones very last time at the very last moment of life. I want to see my daughter and hold my husband’s hand when I die.”* [PLWD, Female, 70–79 years].

Most of the physicians, nurses, and care workers mentioned that the presence of family members is one of the most desired things by the PLWD.

*“It doesn’t matter how good the hospital is, how helpful the staff are, PLWD always wanted to be surrounded by their family in general”* [Nurse, Female, < 50 years].

Nurses and care workers stated the significance of the healthcare facility’s environment, suggesting that frequent family visits can establish a home-like familiar setting. This is crucial because the reluctance of PLWD to be institutionalized often arises from the fear that their families might not be able to meet them regularly.

### Family’s coping with loss

“Family’s coping with loss” was recognized by all participant groups except for the PLWD participants. They, including family members, recognized that family members must cope with the consequences of dementia while playing a crucial role in supporting PLWD. Family caregivers acknowledged the need to learn about the physical and mental changes caused by dementia. They recognized the challenges faced by PLWD in daily activities and the difficulty of expressing wishes due to poor verbal communication skills in advanced dementia. Family caregivers expressed their dedication to provide essential support for their loved ones all the way from diagnosis to death.

*“My husband has dementia, I need to accept it and help him”.* [Family caregiver, Female, 70–79 years]

Physicians, nurses, and care workers acknowledged the dependency of PLWD on their family members for various aspects of care. They recognized that it is vital that families must cope with experiencing the loss, or the consequences of dementia.

*“If anyone has dementia, that person might not be able to do regular activities (both activities of daily living and instrumental activities of daily living) and depend on family members. Therefore, the family has to cope with the consequences.”* [Physician, Male, 50–59 years].

According to physicians, nurses, and care workers, a crucial element of a good death is the care and support for PLWD by family members, including visiting them, participating in their daily care, conversing with them, and understanding, honoring, and fulfilling their wishes. In other words, it is imperative for the family members to cope with gradual loss.

### Maintaining regular life

A good death cannot be defined only by the moment of death. “Maintaining regular life” for PLWD refers to their desire to engage in familiar and preferred activities that bring them joy, emphasizing present experiences and relishing life. The duration of EoL can vary for PLWD, making the performance of preferred activities crucial. Different participant groups highlighted various aspects of “maintaining regular life.“ Namely, PLWD focused on enjoyable daily life until death, and others focused on how to support and enable it.

PLWD stated the importance of having an enjoyable life as death approached. Most of the PLWD participants mentioned the necessity of doing activities they enjoy and having events that give them a sense of an enjoyable life. They hope to die while having a pleasant life instead of dying after a restricted life.

*“I will do my best to enjoy what I like even if I forget it tomorrow*.” [PLWD, Female, 80–89 years].

*“I am not that worried about death, I want to go to bars, watch baseball matches, and do other activities like fishing and hiking.”* [PLWD, Male, 70–79 years].

Notably, PLWD participants realized and indicated that family support is essential to perform activities they enjoy. They also expressed their desire to pass away before reaching the advanced stage of dementia. They understood that at that point, they would need to depend entirely on others and would lose the ability to engage in activities they enjoy.

Participants from other groups recognized the crucial role of engaging PLWD in their preferred activities that they like and enjoy. Moreover, denying PLWD the opportunity to engage in preferred activities impacts their emotional well-being and undermines their sense of dignity. They acknowledged that preferred activities are often individualized and may change over time. Family members, as the primary care providers, play a crucial role in assisting PLWD by engaging them in preferred activities, especially when dementia progresses and PLWD may not be able to maintain regular lives in terms of engaging in preferred activities without their support.

While acknowledging the importance of assisting PLWD in engaging in preferred activities, family caregiver participants, in some cases, diverged in their opinions regarding how best to facilitate and support PLWD in participating in their preferred activities. Most acknowledged that family caregivers have a crucial role in understanding and supporting PLWD in doing their preferred activities; additionally, some mentioned the importance of collaborative engagement involving family caregivers, care workers, and others to enable PLWD to enjoy their desired activities. A few physicians and nurses who talked on this issue focused first on the role of the family and then on collaborative engagement. According to care worker participants, to accomplish a good death of PLWD, addressing their wishes as much as possible, assisting them in performing their preferred activities, and securing family support are important.

### Living with respect

‘Living with respect’ was revealed as an important theme. This points to the desire of PLWD not to be a burden. It also highlighted the perspectives of family caregivers and care workers, that is, the need for treating PLWD in the same as they were treated before being diagnosed with dementia.

PLWD participants wished to die without burdening their families. This sentiment was shared by a majority of PLWD participants who wished to maintain their independence in activities of daily living, doing preferred activities, and avoiding becoming a burden for their loved ones.

*“I try not to depend on others and put a burden on my family and others till death*.” [PLWD, Male, 80–89 years].

Family caregivers also expressed the concern that people with advanced-level dementia may lose their dignity because of physical constraints. They noticed that PLWD often express their desire not to burden their family members. The family caregivers acknowledged the perception of PLWD regarding living with respect is closely connected to their ability to remain independent in activities of daily living, engage in preferred activities without restrictions, and avoid dependence on others. Care worker participants mentioned that PLWD in later stages require assistance in performing activities of daily living, which could impact their sense of dignity. Furthermore, both family caregiver and care worker participants pointed out that individuals with dementia are often ignored and their opinions disregarded.

“*When a person has dementia, I think they are often ignored, especially their opinions, and sometimes they are excluded from everyday conversations*” [Family caregiver, female, **<** 50 years].

Many family caregiver and care worker participants highlighted that if PLWD feel that they are burdensome, it can have negative impacts on their emotional well-being and dignity. Most of them mentioned that PLWD should be treated with respect as before their diagnosis so that they do not feel burdensome, ignored, or undervalued. The support of the family is vital in achieving this, as they are often the primary caregivers, as mentioned by family caregivers and care workers. Physicians and nurses echoed similar statements. They also emphasized that it would be essential to create an environment where family members and others can feel that PLWD are not a burden to them and can value their opinions.

### Preparation for death

Preparing for death, including advance care planning (ACP) and completing unfinished tasks, was found to be an integral component of a good death.

Physicians and nurses emphasized the importance of knowing the treatment preferences of PLWD to respect their wishes and mentioned that both the PLWD and family members need to discuss among themselves and with the physicians about future care preferences. Care worker participants echoed similar statements. When discussing ACP, family caregivers mentioned that family members need to play a key role in ensuring that their loved one’s wishes and values are honored, acting as advocates, and facilitating conversations with healthcare professionals. However, they also mentioned challenges in valuing the preferences of PLWD, especially when medical care for their deteriorating health might take priority. Moreover, according to some family caregivers, it is crucial to wrap up any unfinished business or liabilities before death. Resolving these matters provides a sense of closure and helps alleviate potential burdens for both the PLWD and their family members.

*“There might be some issues like financial or property-related. I know my father’s condition might deteriorate in the future. I would like to discuss whether he has any liabilities in advance. I think it’s not good to die with any financial loan or liabilities*”. [Family caregiver, Female, **<** 50 years]

Surprisingly, none of the PLWD participants talked about aspects that reflect the theme of “preparation for death”, except for one PLWD who mentioned discussing with a family member to avoid a specific treatment procedure.

### Interrelations among themes

All the themes are interrelated. Achieving both painless death and dying in a preferred environment could be influenced by the physical condition of PLWD. Family caregivers indicated that it would be difficult to avoid admitting PLWD if their physical conditions worsen to receive round-the-clock medical care for their loved ones. On the other hand, PLWD participants expressed a desire to remain at home, although they also wanted to have a painless death, specifically, minimal suffering. To balance these possibly conflicting perspectives, the importance of having prior discussions among PLWD, family caregivers, and physicians regarding treatment preferences and preferred places, namely, the importance of ‘preparation for death’, was indicated. The preference of PLWD to die at home or in their preferred environment is also linked to their desire to pass away in the presence of family members, which is also linked to the theme ‘family’s coping with loss’. Furthermore, the family’s coping with loss is central to the other three themes: maintaining regular life, living with respect, and preparation for death. Family members, as primary care providers, play a crucial role in assisting PLWD by engaging them in preferred activities. This becomes particularly crucial as dementia advances, and maintaining their usual engagement in preferred activities may be unattainable without family support. PLWD participants also expressed the crucial role of family support in performing activities they like. The theme of “living with respect” showed the wish of PLWD to not burden others. It also highlighted the viewpoints of family caregivers and care workers, emphasizing the importance of treating PLWD as they were before their dementia diagnosis. Treating them with respect like before their diagnosis prevents them from feeling like a burden, being ignored, or not valued. Family support was mentioned as key in making this happen. ACP and completing unfinished tasks were noted as an integral part of the theme ‘preparation for death’. Family members role in ensuring that their loved one’s wishes and values are honored, acting as advocates, and facilitating conversations with healthcare professionals were considered crucial. Moreover, family members wanted to have a discussion with PLWD about any unfinished business ahead of time. Similarly, the theme “maintaining regular life” is related to the theme “living with respect”, as PLWD may lose their dignity if they are kept from engaging in their preferred activities. Preparation for death that included ACP is directly related to themes, painless death, and dying in a preferred environment.

## Discussion

In this study, we explored the perception of a good death for PLWD from the perspectives of PLWD, family caregivers, care workers, physicians, and nurses. The findings revealed that a good death cannot be considered only in the moment of death but the journey toward death. Participants expressed their views on a good death through a range of elements: painless death, dying in a preferred environment, family’s coping with loss, maintaining regular life, living with respect, and preparation for death. Notably, these themes are interrelated. Not surprisingly, the identified themes could also be applicable to the general elderly population. The fact that some components are conditional on others could be useful for designing and providing care to PLWD to achieve a good death. To our knowledge, none of the earlier studies reported such interrelations among themes.

This study identified two closely related and sometimes incompatible themes: painless death and dying in a preferred environment. This study found painless death to be a core theme, consistent with earlier studies conducted among various participant groups, including the general population, health service providers, and people with cancer [21, 22]. A recent study among physicians and nurses experienced in caring for dying patients with life-threatening illnesses also reported dying without experiencing suffering, both physical and psychological as a principal component of a good death [23]. In our study, while both PLWD and family caregiver participants emphasized achieving a painless death, their perspectives differed. Both groups agreed on the importance of minimal suffering and free from sadness, but they had varying opinions about the use of life-sustaining treatment. PLWD opposed its use, while family members had diverse preferences, including favoring life-sustaining treatments and following physicians’ suggestions. A study conducted in the USA among family members of individuals with cognitive impairment residing in nursing homes revealed mixed preferences for life-sustaining treatments, with some opposing them and others supporting their use [24]. Regarding the theme of ‘preferred environment’, the desire of PLWD to die at home or in their preferred place aligns with previous studies conducted on cancer patients and general populations in the USA, UK, Japan, and China [21, 25–28]. In contrast, family caregivers often prioritized institution-based medical services for PLWD due to their deteriorating physical conditions. Our finding of ‘living with respect’ is also consistent with previous studies, indicating that maintaining dignity or living with respect is a central component of a good death in Japanese society [28–30]. A study in Japan involving nurses who provided EoL care to PLWD reported the importance of valuing their dignity for a good death. The study also highlighted the importance of recognizing their changing physical and cognitive state and accepting them as they are to uphold their dignity [31]. The theme of ‘family’s coping with loss’ emerged as a key aspect of a good death. It was considered a prerequisite for achieving other themes, such as dying at home or in one’s preferred environment, maintaining regular life, and preparing for death. Previous studies in Japan focusing on emergency department patients and cancer patients reported similar findings [30, 32].

Our study identified the theme of ‘maintaining regular life’. Given that PLWD may have varying EoL durations ranging from months to years, the concept of ‘maintaining regular life’ through engagement in preferred activities appears reasonable. In contrast, earlier studies involving family caregivers and care professionals focused on good death for PLWD but did not specifically identify this theme; instead, they highlighted physical health care [16, 18]. The variation in findings could also be attributed to the inclusion of family members of advanced-level PLWD in one study [16] and family members of PLWD who had died within the previous 2–6 months in another study [18]. Consequently, the findings of the present study may not be directly applicable to people living with advanced dementia or family caregivers responsible for the care of PLWD at an advanced stage. Our study showed that physicians, nurses, and care workers stressed the importance of ACP as a part of the theme “preparation for death”. An Australian study involving specialist physicians in palliative and acute settings similarly highlighted the significance of involving patients and families in treatment decisions to achieve a good death [33]. Although an earlier study conducted in the USA among participants in EoL care and their families showed the importance of clear decision-making ability by the dying person [34], the present study did not find such an impression in determining a good death for PLWD. However, in Japanese society, older people often opt to leave decisions on end-of-life care to others [28, 35]. Notably, with the progression of dementia and its associated cognitive decline, the ability for clear decision-making diminishes, particularly in the advanced stages of dementia. A recently published systematic review explored patients’ perception of a good death and found religious activity as a major component from the viewpoint of individuals with AIDS, cardiovascular disease, and cancer [8]. However, these issues remain missed in this study. In general, religious activities have relatively less significance in Japan [36]. A recent study conducted in Japan also reported the absence of religious activities as a primary consideration in the daily life wishes of PLWD [37].

### Implications in research and practice

Our study findings have important implications for the care of PLWD. Physicians and caregivers should proactively ask PLWD about their specific concerns regarding their impending death and their perception of a good death. Given the diverse interpretations people may have, it is important to encourage everyone to express their own understanding of a good death. Preferences for a good death may change over time, emphasizing the need to understand what matters most to individuals at different stages of their disease. This understanding is crucial for ensuring a good death.

Conflicting perspectives between PLWD and their family members may arise, but the objective should be to find a solution that respects the opinion of PLWD while considering the practical aspects of their care. Our findings underscore the importance of ACP, particularly regarding making decisions in advance about future treatments and care preferences. Initiating ACP discussions soon after a dementia diagnosis is vital to honoring the individual preferences of PLWD.

Our findings could help in developing a Good Death Inventory (GDI) for PLWD. The GDI was developed and used in Japan to investigate how bereaved family members of cancer patients perceive a good death [38] and was also employed in China to understand preferences for a good death among cancer patients [39]. We could use a similar approach for PLWD, using Likert scales to measure aspects of a good death. This could be used at different stages of dementia. Future studies should validate the GDI for PLWD, and assess the effectiveness and utility of such tools for achieving good death. The GDI could serve as a comprehensive assessment tool to identify preferences for a good death from the perspective of both PLWD and family members. By assessing these perspectives, we could gain insights into the similarities and differences in their preferences and facilitate more effective decision-making and care planning for dementia. Furthermore, it could assist healthcare providers in identifying areas where support and interventions are needed. However, to our knowledge, no relevant investigation in PLWD has been conducted to date.

### Strengths and limitations

The key strength of this study is the inclusion of PLWD and multiple stakeholders involved in dementia care. The robustness of the methods, including triangulation, peer debriefing, and member checking, is another strength of this study. However, this study has some limitations. Only noninstitutionalized PLWD were included in this study. The present findings may not be directly applicable to institutionalized PLWD or those with a severe/advanced stage of dementia. Additionally, none of the participants with dementia were living alone. The views of PLWD living alone and institutionalized PLWD, or those in a severe/advanced stage of dementia, should be studied, as they may differ from those of the studied group. In this study, physicians from dementia outpatient clinics and daycare center managers helped to identify potential participants. We interviewed those who were interested and gave consent to participate; therefore, there might be a selection bias. However, given the diversity of our study participants, we believe they represent a broad variety of stakeholders. The study was conducted in Japan, and the findings may not be generalizable to other cultural contexts.

## Conclusion

In this study, we explored the perceptions of a good death for PLWD. The findings highlight painless death, dying in a preferred environment, family’s coping with loss, maintaining regular life, living with respect, and preparation for death. This study also highlights the different opinions among stakeholder groups. It is important to give special attention to ACP after the diagnosis of dementia.

### Electronic supplementary material

Below is the link to the electronic supplementary material.

Supplementary Material 1

## Data Availability

The data generated and analyzed during the current study are not publicly available due to privacy issues but are available from the corresponding author on reasonable request.

## Abbreviations

ACP: Advance Care Planning
EoL: End-of-Life
FGD: Focus Group Discussion
GDI: Good Death Inventory
IDI: In-depth Interview
PLWD: People Living with Dementia
WHO: World Health Organization
