# Supplementary material for: Good death for people living with dementia: a qualitative study
Source: BMC Geriatr. 2023 Oct 16;23:665. doi: 10.1186/s12877-023-04395-y (PMC10580641; doi:10.1186/s12877-023-04395-y)
Supplement: Supplementary file 1 — Supplementary Material 1 [file 12877_2023_4395_MOESM1_ESM.docx]

Topic guide for IDI

Part 1: General information

- Age:
- Gender: □ Female □ Male

Participant Group: □ PLWD □ Physician □ Nurse

Part 2 – Questions to explore

- Please share your thoughts on what constitutes a "good death"?
- How a good death can be achieved?
- Which components are important to achieve good death?
- Please explain the reasons behind your opinion?
- Who can play role in achieving good death for PLWD?
- Do you have any additional opinion regarding a good death for PLWD?
- Are there any concerns or areas you feel need improvement in ensuring good death?

Note: Ask probing question to get greater insight of initial response, where necessary.

Topic guide for FGD

Part 1: General information

- Collect age and gender information of all participants.
- Number of participants

Part 2 – Questions to explore

- Please share your thoughts on what constitutes a "good death"?
- How a good death can be achieved?
- Which components are important to achieve good death?
- Please explain the reasons behind your opinion?
- Who can play role in achieving good death for PLWD?
- Do you have any additional opinion regarding a good death for PLWD?
- Are there any concerns or areas you feel need improvement in ensuring good death?

Note: Ask probing question to get greater insight of initial response, where necessary.
